# The Spleen as an Optimal Site for Islet Transplantation and a Source of Mesenchymal Stem Cells

**DOI:** 10.3390/ijms19051391

**Published:** 2018-05-07

**Authors:** Naoaki Sakata, Gumpei Yoshimatsu, Shohta Kodama

**Affiliations:** 1 Department of Regenerative Medicine and Transplantation, Faculty of Medicine, Fukuoka University, Fukuoka 814-0180, Japan; naoakisakata@fukuoka-u.ac.jp; 2 Center for Regenerative Medicine, Fukuoka University Hospital, Fukuoka 814-0180, Japan; gyoshimatsu@fukuoka-u.ac.jp; 3 Research Institute for Regenerative Medicine, Fukuoka University, 7-45-1 Nanakuma, Jonan-ku, Fukuoka 814-0180, Japan

**Keywords:** spleen, islet transplantation, transplant site, immunity, tolerance, regeneration, diabetes mellitus, mesenchymal stem cell, Sjogren’s syndrome, HOX

## Abstract

This review demonstrates the unique potential of the spleen as an optimal site for islet transplantation and as a source of mesenchymal stem cells. Islet transplantation is a cellular replacement therapy used to treat severe diabetes mellitus; however, its clinical outcome is currently unsatisfactory. Selection of the most appropriate transplantation site is a major factor affecting the clinical success of this therapy. The spleen has long been studied as a candidate site for islet transplantation. Its advantages include physiological insulin drainage and regulation of immunity, and it has recently also been shown to contribute to the regeneration of transplanted islets. However, the efficacy of transplantation in the spleen is lower than that of intraportal transplantation, which is the current representative method of clinical islet transplantation. Safer and more effective methods of islet transplantation need to be established to allow the spleen to be used for clinical transplantation. The spleen is also of interest as a mesenchymal stem cell reservoir. Splenic mesenchymal stem cells contribute to the repair of damaged tissue, and their infusion may thus be a promising therapy for autoimmune diseases, including type 1 diabetes mellitus and Sjogren’s syndrome.

## 1. Introduction

The spleen is located in the left upper quadrant of the abdomen. It is a peripheral lymphoid organ with an important role in the immune system, including in the maturation of B cells, T cells and plasma cells, and the production of immunoglobulin M [1]. The spleen also acts as a hematopoietic organ during the prenatal period and in the event of massive hemorrhage and bone marrow deficiency, and acts to remove old red blood cells and as a blood reserve. Nevertheless, the spleen has frequently been considered as an “unnecessary organ” because its functions can also be carried out by other organs, and loss of the spleen does not directly lead to death. Splenectomy has therefore been widely performed in patients with splenic injuries or pancreatic malignant tumors. However, overwhelming postsplenectomy infection has recently been recognized as a severe complication of splenectomy, associated with high mortality. Furthermore, loss of the spleen causes immunological hypofunction, leading to exacerbation of bacterial infections involving *Streptococcus pneumoniae*, *Haemophilus influenzae* and *Neisseria meningitides* [2], thus emphasizing the importance of this organ.

The importance of the spleen is not limited to its immunological properties [3]. In this review, we demonstrate the unique potential of the spleen as an optimal site for islet transplantation and as a source of mesenchymal stem cells.

## 2. Islet Transplantation and Its Obstacles

Islet transplantation is a cellular replacement therapy used to treat severe diabetes mellitus in patients who are unable to control their blood glucose levels, even with intensive insulin treatment. Islet transplantation enables patients to receive an appropriate supply of insulin in response to changes in blood glucose levels, and can help to prevent severe hypoglycemia and life-threatening complications, including cardiomyopathy, nephropathy, retinopathy and neuropathy [4,5,6]. Islet transplantation was first established in the clinic in the 1970s [7], but early therapeutic outcomes were inadequate, and islet transplantation is still regarded as an experimental therapy. At the end of the 1990s, fewer than 50% of patients achieved insulin-independence two months after islet transplantation and fewer than 10% after a year [8]. However, the development of an automated method of islet isolation in the mid-1980s represented a turning point in islet transplantation. This method involves the progressive chemical and mechanical digestion of the pancreas in warm collagenase solution using a digestion chamber known as a Ricordi chamber [9]. The islets are then purified from the digested pancreatic tissue by density-gradient separation using a blood cell processor (IBM 2991 device, sold as COBE 2991^®^; Terumo BCT, Inc., Lakewood, CO, USA). These advances in digestion and islet purification techniques have enabled large numbers of islets to be harvested with high purity, and contributed to the first clinically successful islet transplantation at Washington University in St Louis in 1989. This was performed in a 36-year-old woman with type 1 diabetes mellitus, who received transplantation of approximately 800,000 islet equivalents and who subsequently achieved normoglycemia for 22 days without insulin treatment [10].

Another turning point was the development of an effective immunosuppressive regimen for islet transplantation. In 1990, a group in Pittsburg successfully prolonged insulin independence for over three months in a clinical allogeneic islet transplantation study using tacrolimus (FK506) [11]. Tacrolimus is a calcineurin inhibitor that is required for T-cell receptor induction of interleukin-2 (IL-2) and for T cell proliferation. It has a superior safety profile compared with the earlier calcineurin inhibitor, cyclosporine [12,13]. At the end of 1990s, an Edmonton group developed an islet transplantation protocol using the steroid-free immunosuppressive agents sirolimus, daclizumab and tacrolimus, and reported on seven patients with severe type 1 diabetes who were all able to function without insulin treatment with no episodes of hypoglycemic coma [14]. Sirolimus (rapamycin) inhibits the activation of T and B cells by suppressing the multifunctional serine-threonine kinase, mammalian target of rapamycin, which is required for efficient production of IL-2 [15,16]. Daclizumab is a monoclonal antibody directed against CD25, a component of the IL-2 receptor, which blocks the formation of the high-affinity IL-2 receptor and can prevent acute rejection by inhibiting the expansion of cytotoxic T cells [17]. The currently recommended protocol includes antithymocyte globulin plus the recombinant soluble tumor necrosis factor receptor protein, etanercept, as induction immunosuppressant agents, followed by tacrolimus or cyclosporine plus mycophenolate mofetil (an inhibitor of purine biosynthesis) for immunosuppression maintenance. A group from Minnesota tested this protocol in six recipients and showed that four of them became insulin-independent for a mean of three years [18].

Novel immunosuppressant regimens are being developed with dramatic speed. In a recent study, human glioblastoma was successfully engrafted for two months in mice administered four treatments of cytotoxic T-lymphocyte-associated protein 4 immunoglobulin and anti-CD154 antibody [19], which induced the immune tolerance required to achieve long-term xenotransplantation. Although this was not an islet transplantation study, it nevertheless demonstrated that it was possible to achieve xenotransplantation, including islet transplantation, by further improvement of immunosuppressants.

The outcome of clinical islet transplantation has improved dramatically over the past 50 years due to technological improvements. A report in 2005 by the Edmonton group, analyzing the long-term outcomes of their 65 patients, showed that approximately 80% of them achieved successful islet engraftment at five years after transplantation (i.e., detection of serum C-peptide and reactivity to glucose stimulation), although only 10% remained free from insulin treatment [20]. A recent report from the Collaborative Islet Transplant Registry (a registry of clinical islet transplant cases performed in USA, Europe or Australia) indicated that rates of insulin-independence at three years after transplantation have been improving, from 27% in 1999–2002 to 44% in 2007–2010. Positive fasting C-peptide levels (≥0.3 ng/mL) were also significantly higher in the period 2007–2010 compared with from 1999–2002 (90% vs. 60% at three years after transplantation) [21]. Moreover, approximately 80% of recipients who received ≥600,000 total islet equivalents achieved insulin independence, compared with 55% who received <600,000 islet equivalents [22]. Islet transplantation is therefore now considered to be a practical option for treating patients with severe diabetes mellitus to improve endocrine function and prevent hypoglycemic attack; however, clinical outcomes currently remain unsatisfactory. Key requirements for a positive outcome include the acquisition of large numbers of islets from the donor pancreas, prevention of graft loss in the early stage of transplantation and maintaining engraftment for a long period. The transplant site is also an important factor influencing engraftment, and the outcome of clinical islet transplantation could be further improved by utilizing a more suitable transplant site.

## 3. Candidate Transplantation Sites for Islets

The optimal islet transplantation site should meet the following criteria: (1) an abundant, oxygen- and nutrient-rich blood flow; (2) be privileged immunologically to minimize transplant graft loss; and (3) allow transplantation with minimal invasiveness. Numerous organs have been assessed to date, including the liver [23,24,25], renal subcapsular space [23,24], omental pouch [26,27], mesentery [28], gastrointestinal tract [29], skeletal muscle [30], subcutaneous tissue [30], eye [31], brain [32], testis [33,34], bone marrow [35], thymus [36], and spleen [37]. However, it has been difficult to find a site that meets all three criteria (Table 1).

The liver has long been used as a site for clinical islet transplantation. It is the largest organ and can thus accommodate large numbers of islets following a simple transplant procedure (percutaneous infusion into intrahepatic portal vein using ultrasonography under local anesthesia) [38]. However, transplantation into the liver is also associated with some problems. Many islets are destroyed in the early stages of transplantation, partly due to hypoxia caused by ischemia. The isolated islets are in an avascular state throughout the process of preparation [39] and are exposed to hypoxia in the hypo-oxygenized portal venous blood (mean PO_2_ approximately 5 mmHg [40]) until revascularization occurs. Moreover, the islets themselves can cause liver ischemia by embolizing the peripheral portal vein [41,42]. Inflammation and immunity may also be issues. The transplanted islets are frequently the subject of an innate immune response and are attacked by tissue macrophages in the liver known as Kupffer cells [43,44], and by natural killer cells [45], which may in turn induce an adaptive immune response. Furthermore, infusion of islets into the blood stream can trigger an instant blood-mediated inflammatory reaction (IBMIR), which can damage intraportal transplanted islets [46]. The IBMIR is triggered by the exposure of islet surface molecules, such as tissue factor (coagulation factor III), during islet isolation and purification [47,48]. Tissue factor causes rapid binding of platelets, leading to coagulation and complement system activation. Most of the islets are destroyed by this reaction within 1 h after transplantation [47]. Moreover, some immunosuppressants can be more toxic to islets in the liver because they are present in higher concentrations in the portal vein than in peripheral vessels [49]. Other complications of intraportal islet transplantation include portal hypertension and portal thrombosis. Portal hypertension can increase the risk of post-transplant bleeding, portal thrombosis and sepsis [50,51], while portal thrombosis is a life-threatening complication of islet transplantation, potentially leading to esophageal varices, splenomegaly, mesenteric ischemia, sepsis and death [52].

The kidney (i.e., renal subcapsular space) has frequently been used for islet transplantation in experimental studies, especially in rodents, and islet transplantation into the kidney has been reported to restore normoglycemia. However, these studies used relatively small numbers of islets because the inelastic and tight nature of the human renal subcapsular space makes it is difficult to transplant large numbers of islets [53]. This may explain why clinical progress in renal subcapsular islet transplantation has lagged behind other techniques [54].

Muscle and subcutaneous tissues have also been examined as candidate transplantation sites, given that the transplantation procedure and biopsies can be performed easily with minimal invasion and few complications. However, these sites suffer from hypovascularity and hypoxia, and the transplantation efficacy, especially in subcutaneous tissue, could be improved if these obstacles were overcome [30]. These sites also have the disadvantage of systemic insulin release. In general, secreted insulin from the pancreas flows into the liver via the portal vein, and smaller amounts of insulin are therefore needed to control blood glucose. This is referred to as physiological insulin secretion, compared with systemic insulin release. Islet transplantation into intramuscular and subcutaneous sites results in systemic insulin release, which requires the release of much larger amounts of insulin, similar to those required by insulin injection therapy, because the insulin does not enter the portal system directly.

Another favorable islet transplantation site is the omental pouch. This site has the advantages that its insulin drainage occurs via the portal vein, making it closer to the physiological situation, and the site is highly vascularized [55]. There has been much progress in intra-omental pouch islet transplantation in rodent [27], dog [56] and nonhuman primate models [57]. Because the omental pouch is highly vascularized, it has been proposed as an alternative site for encapsulated islet transplantation [58,59,60], but no clinical trials have been performed to date.

The mesentery is also considered as a candidate islet transplant site due to its rich vascularization and ability to accommodate many islets. However, it has the disadvantage that, in the event of any problem with the graft, it would be difficult to remove it without damaging the intestinal tract [61]. The submucosal space of the gastrointestinal tract is also a possible transplant site, with a rich vascular supply providing oxygen and nutrients, and which connects to the same portal system as the liver, spleen and pancreas [55]. Although Hara and colleagues studied transplantation into this location by endoscopy in a pig model [29,62], there have been limited demonstrations of this concept in large animal models.

The brain, testis, anterior chamber of the eye and thymus are organs where the immunological response is suppressed and are thus considered as “immune privileged” sites. This immune privilege was once assumed to be due to a lack of cellular infiltration and lymphatic drainage [63], but it has recently been shown that it is provided by a complex of immune responses [64]. For example, the brain, testis and retina–blood barrier are maintained in an immunosuppressed condition due to a cellular physical shield [64,65,66], while regulatory T cells (Tregs) also contribute to immune privilege in some cases. Larocque and colleagues showed that the immune response in the brain could be activated normally when CD4+CD25+ Tregs were depleted [67], while Hedger further revealed that rodent testes contained significant numbers of immunoregulatory cells, including Tregs [68]. Farooq et al. recently showed that Tregs contributed to immune tolerance in the anterior chamber in rodents when challenged by myelin antigen [69]. Many experimental trials have investigated allo- and xenogeneic islet transplantation into immune privileged sites in non-human animals. However, although such studies have demonstrated the effectiveness of transplantation into immune privileged sites [31,32,34,36], little has been done in a human clinical setting, and the brain or eye are considered particularly problematic sites for transplantation, because it would be difficult to remove a graft in the event of graft failure without damaging the transplanted organ.

## 4. Characteristics of the Spleen as an Islet Transplant Site

Among the above candidate islet transplant sites, the spleen may represent the optimal site. It is a highly vascularized organ that receives blood from the splenic artery and drains into the portal venous system. Given that vascularization is the most important factor determining the success of transplantation, the spleen provides a rich oxygen and nutrient supply. Furthermore, islets transplanted into the spleen can achieve physiological levels of insulin secretion, given that insulin produced by pancreatic β cells flows into the portal–splenic vein (portal venous circulation) [70]. In contrast, insulin provided by a subcutaneous pump or by injection is delivered directly into the systemic circulation. Although recent advances in these insulin injection systems enable them to achieve close to physiological insulin release profiles (i.e., in the portal system), they remain limited by day-to-day changes in insulin sensitivity [71]. Because the spleen connects to the portal venous system, like the liver and pancreas, insulin released from islets transplanted in the spleen flows into the splenic vein.

The spleen is responsible for immune tolerance and thus tends to be immunosuppressed, though to a lesser extent than immune privileged sites such as the testis and thymus. Previous studies have revealed that the spleen is involved in the suppression of T cell proliferation and antibody production following the induction of immune tolerance [72,73], while other studies have shown that splenic dendritic cells are a good source of suppressor cytokines, including transforming growth factor-β. The splenic T cell population was shown to include suppressor T cells [74], also known as Tregs [75]. Tregs in the spleen prevent antigen presentation by dendritic cells to effector T cells, and suppress proliferation of effector T cells via production of suppressor cytokines including transforming growth factor-β, IL-10 and IL-35 [76]. Horton and colleagues performed intrasplenic allo-transplantation of islets into lymphoid-irradiated dogs that had received donor bone marrow transplantation before transplantation. They observed that islet graft function was maintained after total pancreatectomy without the use of immunosuppressants [77]. Moreover, splenocytes themselves may help regulate autoimmunity. We previously rescued non-obese (NOD) mice (representative type 1 diabetes mellitus animal model) from a severe diabetic condition by injection of live donor splenocytes with complete Freund’s adjuvant to eliminate autoimmunity. In contrast, NOD mice that received irradiated splenocytes all became diabetic. Immune attack against lymphoid cells was minimal when live splenocytes were injected into complete Freund’s adjuvant-infused mice [78,79]. It is therefore not surprising that the spleen can also protect transplanted islets from innate inflammatory responses, which are a major factor contributing to islet graft failure, together with acquired immune responses. We previously reported that several kinds of inflammatory cytokines, including monocyte chemotactic protein-1, granulocyte-colony stimulating factor, and high-mobility group box 1 (HMGB1), were increased in the plasma after intraportal islet transplantation [80,81,82]. We also confirmed that levels of these cytokines were significantly lower in intrasplenic compared with intraportal transplantation [83].

Interestingly, the spleen has been shown to be a reservoir of islet stem cells in diabetic mice (see below). We confirmed that CD45− (nonlymphoid) splenocytes could develop into stem cells and further differentiate into islet progenitor cells, thus contributing to islet regeneration [78]. Moreover, we found that adult mice spleens contained putative mesenchymal stem cells expressing Hox11 (Tlx1, a marker of splenic stem cell [84]) but not Pdx1 (an early pancreatic regeneration marker), which were CD45− in origin [85]. Lee and colleagues provided additional evidence showing that removal of the spleen in children with severe thalassemia led to the eventual development of insulin-dependent diabetes [86]. The spleen may thus facilitate the proliferation of intrasplenic transplanted islets. In 1989, Wohlrab and colleagues first observed the proliferation of β cells in intrasplenic transplanted islets at 200 days post-transplantation. They speculated that the proliferative response resulted from long-term stimulation by slightly enhanced plasma glucose levels at the transplantation site [87]. We also observed proliferation of intrasplenic islets transplanted into the renal subcapsule, and these transplanted islets expressed both insulin and ribonucleoside-diphosphate reductase subunit M2 b (Rrm2b) [83]. The Rrm2b gene encodes the small subunit of a p53-inducible ribonucleotide reductase and plays a role in DNA synthesis, and its expression may therefore contribute to proliferation of the transplanted islets [88].

**Figure 1:**
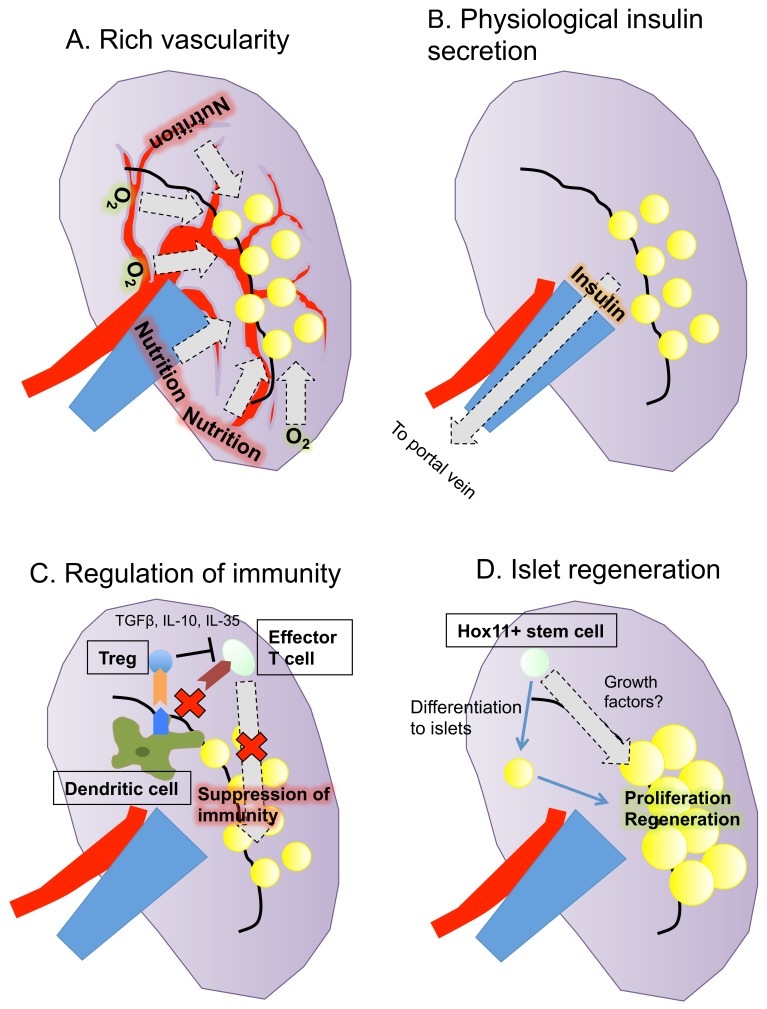
Summary of the characteristics of the spleen as a transplantation site for islets. The spleen has four advantages as a site of islet transplantation: (**A**) rich vascularity; (**B**) physiological insulin secretion; (**C**) regulation of immunity; and (**D**) potential for islet regeneration.

In summary, the spleen may be close to being an optimal site for islet transplantation because of its rich vascularity, physiological insulin secretion, regulation of immunity including autoimmunity and its potential for islet regeneration (Figure 1).

## 5. Outcomes of Intrasplenic Islet Transplantation

The major studies on intrasplenic islet transplantation are summarized in Table 2. Historically, intrasplenic islet transplantation has been performed since the late 1970s, when several trials of intrasplenic islet autotransplantation into pancreatectomized dogs demonstrated recovery of endocrine function [89,90,91,92]. This model has been used to assess transplantation efficacy [89,90,92,93,94,95,96,97,98,99,100,101,102], and also to assess the transplantation of cold-preserved or cryopreserved islets [103,104,105,106] and the toxicity of immunosuppressants [95,107,108,109,110]. Other animals, including pigs [111] and monkeys [112,113,114], have also been used for islet autotransplantation and have shown acceptable outcomes.

Some groups in the 1980s used allo- [115] and xenogeneic [116] islet transplant models. Du Toit and colleagues performed intrasplenic allogeneic islet transplantation in pancreatectomized dogs treated with cyclosporine, and showed that survival was extended in comparison with non-immunosuppressed dogs [115]. Moreover, the usefulness of rapamycin in transplantation was also demonstrated in an allogeneic transplant dog model [117]. Andersson reported the survival of allogeneic grafts from cultured islets for several weeks without the use of any immunosuppressants [118]. In a xenograft model, the Washington group succeeded in prolonging graft survival for more than 100 days using cultured islets in a rat-to-mouse transplant model in which the recipients were treated with anti-rat and/or anti-mouse lymphocyte sera [116]. These findings demonstrated the possibility of using the spleen for the transplantation of allo- and xenogeneic islets.

However, although the spleen has many advantages over other transplant sites, the efficacy of transplantation has been somewhat unclear. For example, Evans and colleagues showed that the transplantation efficacy into the spleen was better than that into the liver or kidney in an islet autotransplantation dog model: 90% of animals achieved normoglycemia at one month for the spleen compared with 33% for liver and 0% for kidney [96] (Table 3). Using fetal porcine allotransplantation and murine transplantation models, Stokes et al. showed a higher transplantation efficacy for the spleen compared with the liver, although the kidney was better [129,130]. Many other studies have reported superiority of the spleen compared with the liver [100,111] or omental pouch [56,131], although some groups have reported opposite results [97,98,99] (Table 3).

The route of transplantation into the spleen also needs to be considered. The earliest studies transplanted islets into the spleen pulp [90,92]. However, Warnock et al. tested intrasplenic islet transplantation via the splenic vein in an islet autotransplanted pancreatectomized dog model, and observed greater efficacy versus transplantation into the pulp, achieving normoglycemia in 86% vs. 33% of animals [122] (Table 2). Although the intravenous route has been considered to show greater efficacy for intrasplenic transplantation than the intrapulp route, the intravenous route can increase the risk of IBMIR, which can damage the transplanted islets, similar to intraportal transplantation into the liver [55].

**Figure 2:**
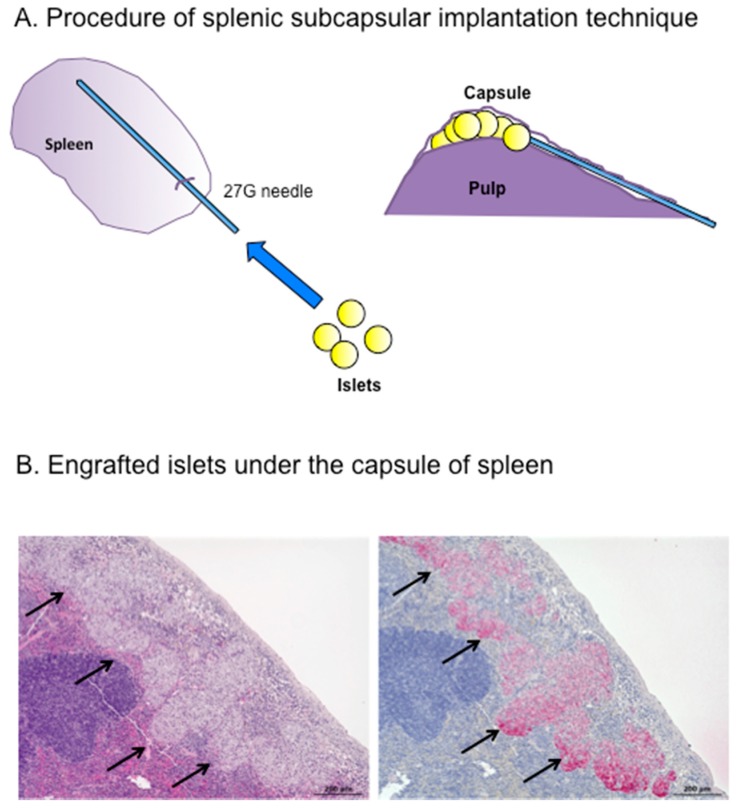
(**A**) Splenic subcapsular implantation technique; and (**B**) Engrafted islets (d arrows) under the spleen capsule 28 days after transplantation ((**Left**) hematoxylin and eosin staining; and (**right**) immunostaining for insulin). Scale bar 200 μm.

To examine the potential usefulness of the spleen as an islet transplantation site and to try to develop a better procedure for intrasplenic transplantation, we explored the “splenic subcapsular implantation technique” using a rodent syngeneic transplant model, and analyzed the transplant efficacy of this method compared with intrahepatic and renal subcapsular transplantation [83]. This procedure involved direct puncture from the surface with a 27-gauge needle and implantation of islets under the splenic surface without venous or pulp injury (Figure 2).

Notably, all the transplanted mice (*n* = 10) achieved normoglycemia for two months, even after intrasplenic transplantation of only 50 islets. In contrast, none of the mice transplanted with islets into the liver or kidney achieved normoglycemia. Intrasplenic transplantation was thus not only superior to other sites in terms of transplantation efficacy, but also allowed three to four diabetic mice to be treated using islets from a single donor mouse (i.e., 150–200 islets can be harvested from one donor mouse). Normoglycemia could also be achieved using as few as 25 islets transplanted into the spleen if glucose levels were also managed rigorously. Histological assessment revealed that the intrasplenic transplanted islets were enlarged in size. The transplantation efficacy of this model clearly exceeded those of previously reported intrapulp and intravenous transplantation models [99,120]. We speculated that this might be because intrapulp and intravenous transplantation involve greater tissue damage and consequent exposure of islets to the blood compared with intrasplenic transplantation, thus inducing IBMIR. In addition to preventing graft loss, intrasplenic transplantation allows the engrafted islets to access a rich oxygen and nutrient supply associated with the abundant blood flow. These factors, together with the privileged immune status of this site, may explain the greater success of engraftment and regeneration.

The spleen has a hard elastic capsule, like the kidney [132]. Although this can be an obstacle in terms of the number of islets it is able to contain, we suggest that this cannot be a major limitation. In contrast to the kidney, the spleen has a softer parenchyma, which provides sufficient space to accommodate many islets, and the hardness of the capsule is therefore not a limitation of intrasplenic islet transplantation using the splenic subcapsular implantation technique.

Regarding the possibility of an intraarterial approach for intrasplenic islet transplantation, Wang and colleagues recently assessed the therapeutic effects of transarterial chemoembolization in patients with unresectable hepatocellular carcinoma using transcatheter intraarterial perfusion magnetic resonance imaging, and demonstrated the usefulness of the imaging examination for showing changes in tumor perfusion [133]. This methodology has three merits in terms of its application to islet transplantation: the transcatheter procedure can be performed under local anesthesia; the target organ for islet transplantation can be selected by the transcatheter procedure (intrasplenic islet transplantation can be done by cannulation of the catheter into the splenic artery); and the condition of the islets after transplantation can be assessed by intraarterial perfusion magnetic resonance imaging. Although there have been no reports of intraarterial islet transplantation to date, it may become an option for islet transplantation, with future modifications.

## 6. Future Clinical Intrasplenic Islet Transplantation

The first intrasplenic islet transplantation was performed in a clinical setting at the University of Leicester 20 years ago. Five chronic pancreatitis patients underwent spleen-preserving total pancreatectomy and intrasplenic islet autotransplantation, of whom two achieved insulin independence for over a year. However, this procedure was associated with high morbidity, including splenic infarction and portal thrombosis [37]. Du Toit et al. reported that intrasplenic islet transplantation was accompanied by life-threatening complications, including subcapsular hematoma, intrasplenic necrosis and cavitation, capsular perforation and arteriolar thrombosis [115]. However, we believe these complications could be overcome with advances in surgical procedures. In our opinion, implantation into the splenic subcapsular region may minimize the risk of necrosis, thrombosis and hemorrhage by preventing venous and pulp injury. Laparoscopic surgery or intraportal transplantation could also minimize the surgical stress of the transplantation procedure. We suggest that, using a combination of the techniques described here, intrasplenic transplantation may offer the optimal approach to islet transplantation.

The spleen has historically been an important site for islet transplantation, but its utility could be greatly improved by the application of recent novel findings and techniques, and we therefore advocate the development of clinical methods to optimize the safe and effective transplantation of islets into the spleen.

## 7. Spleen as a Source of Mesenchymal Stem Cells

**Figure 3:**
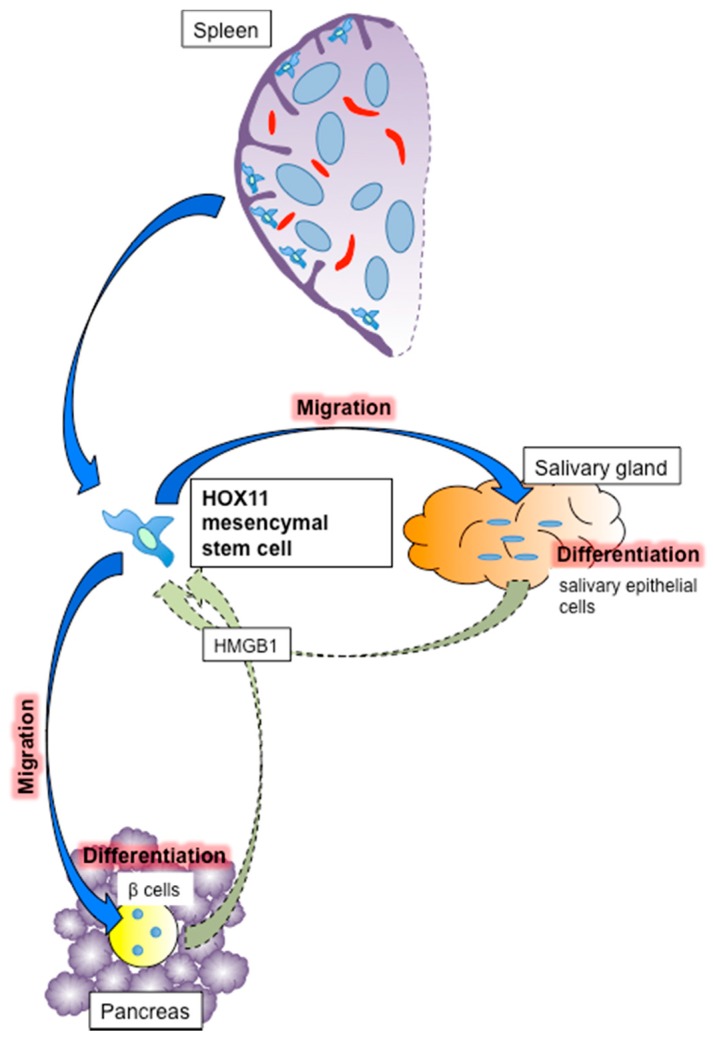
Mechanism of tissue repair by splenic mesenchymal stem cells. Splenic mesenchymal stem cells are normally located under the splenic capsule and migrate to the damaged tissue following stimulation by HMGB1. They then differentiate into tissue components such as β cells in pancreatic islets and salivary epithelial cells in salivary glands. HOX11-positive cells are considered to be splenic mesenchymal stem cells.

We also considered the importance of splenic mesenchymal stem cells. As noted in Section 4, the spleen acts as a stem cell reservoir as well as an islet transplant site. The spleen harbors both hematopoietic and mesenchymal stem cells, with and without CD45 labeling, respectively. CD45− splenic mesenchymal stem cells play an important role in the repair of damaged tissues, as in other organs including the bone marrow and adipose tissue. The first step of tissue repair involves the migration of mesenchymal stem cells specifically into the damaged tissue. Mesenchymal stem cells are believed to detect signals released from the damaged tissue, which cause them to migrate and accumulate selectively in the damaged tissue. The representative signal is HMGB1, which interacts with the nucleosome, transcription factors and histones. HMGB1 influences the structure and remodeling of chromatin by binding to its internucleosomal linker regions to facilitate nucleosome sliding [134]. HMGB1 released from damaged and apoptotic tissues has been shown to activate nuclear factor-kappa B by binding to toll-like receptor 4 and receptor for advanced glycation endproducts expressed on the surface of immune and inflammatory cells, subsequently causing an immune response and inflammatory reaction, and removing damaged and apoptotic tissues [135]. This sequential reaction requires binding between HMGB1 and damaged-tissue-derived DNA and histone protein [136]. Meanwhile, free HMGB1 released from the damaged tissue promotes the migration of mesenchymal stem cells to the damaged tissue and tissue repair [137] (Figure 3). The migration of splenic stem cells is considered to be controlled by HMGB1, especially in the case of inadequately functioning bone marrow due to disease [138].

Following migration to the damaged tissue, splenic mesenchymal stem cells differentiate into the cellular components of the damaged tissue. We previously showed that these cells differentiated into islets [78] and salivary epithelial cells [139]. Regarding islets, syngeneic CD45− splenic stem cells infused into NOD mice migrated into islets damaged as a result of autoimmunity and differentiated into insulin-positive β cells [78] (Figure 3). Robertson and colleagues also showed that dissected quail splenic tissue (presumably including mesenchymal stem cells) differentiated into insulin-producing cells under co-culture with chick pancreatic epithelium [140]. Regarding the salivary gland, we showed that infused splenocytes migrated into damaged salivary glands in NOD mice, as an animal model of Sjogren’s syndrome [141], and differentiated into salivary epithelial cells [139] (Figure 3). Sjogren’s syndrome is an autoimmune disease that destroys salivary and lachrymal glands, resulting in dry eyes and mouth, which symptoms drastically impair patient quality of life. Dry eyes can lead to vision problems including loss of light sensitivity, blurred vision and corneal damage, while dry mouth induces dental caries and oral infection. Tissue repair of the salivary glands can prevent these symptoms and thus improve quality of life, and mesenchymal stem cells may thus be a promising therapy for Sjogren’s syndrome. A novel clinical trial of mesenchymal stem cell infusion therapy was recently applied in Sjogren’s syndrome patients [142]. Given the lack of radical new treatments for Sjogren’s syndrome, mesenchymal stem cell infusion therapy may offer a promising therapeutic strategy for these patients.

CD45− splenic stem cells express OCT3/4, SOX2, KLF4, c-MYC and NANOG proteins, which induce matured cells into induced pluripotent stem cells [84], as well as transcription factors also identified in embryonic stem cells, including HOX11. HOX11 (TLX1), similar to other HOXA subgroups including HOX11L2 (TLX3), is an oncogene that induces T-cell acute lymphoblastic leukemia (T-ALL) by chromosomal translocation of t(10;14)(q24;q11) and t(7;10)(q35;q24) [143]. According to Cancer Research UK, the prognosis of T-ALL is relatively good (five-year survival rate after diagnosis 70%; http://www.cancerresearchuk.org/about-cancer/acute-lymphoblastic-leukaemia-all/survival). Notably, Ferrando and colleagues revealed that the survival rate of HOX11-positive T-ALL patients was significantly better than that of other T-ALL patients (88% vs. 56%, *p* = 0.019) [144]. This suggests that HOX11 is a risk factor for T-ALL, but can also be used as a marker for evaluating patient prognosis. HOX11 is an embryonic protein that contributes to embryonic development, including cell survival, differentiation and regeneration [145,146]. HOX11-positive mesenchymal stem cells are located under the splenic capsule rather than in the pulp of the spleen [84] (Figure 3), and HOX11 is expressed specifically in CD45− splenic mesenchymal stem cells, and not in stem cells from other organs, including the bone marrow and liver [84,85]. HOX11-positive cells are considered to be a major component of the CD45− splenic mesenchymal stem cells that contribute to tissue repair of damaged islets and salivary glands in our models.

The main disadvantage associated with the clinical application of splenic mesenchymal stem cells is the cumbersome nature of the procedure compared with bone marrow or adipose mesenchymal stem cells. Splenectomy is essential for acquiring mesenchymal stem cells, but it is difficult to collect donor spleen. Although splenectomy is indicated for pancreatic and splenic malignant diseases, hematologic diseases such as idiopathic thrombocytopenic purpura and injury, malignant diseases are excluded as indications. Moreover, splenectomy should not be carried out in healthy donors because of the need for laparotomy or laparoscopic surgery under general anesthesia, and potential complications including overwhelming postsplenectomy infection. However, one possible use of splenic mesenchymal stem cells may be autotransplantation in patients with benign diseases, such as injury. Cloning and banking of splenic stem cells is also a promising method for utilizing the cells as required.

## 8. Conclusions

In this review, we demonstrate the importance of the spleen as an optimal site for islet transplantation and as a source of mesenchymal stem cells. Although the spleen has been considered as an unnecessary organ, it has many unique potentials in experimental and clinical medicine. Further studies are needed to clarify these potential applications of the spleen.

## Figures and Tables

**Table 1:** Candidate islet transplantation sites other than the spleen.

Transplant Site	Advantages	Disadvantages
**Liver**	✓ Representative site for clinical transplantation ✓ Relatively easy to access ✓ Physiological insulin secretion	✓ Instant blood-mediated inflammatory reaction ✓ Innate immunity ✓ Portal thrombosis and hypertension
**Kidney**	✓ Highest transplant efficacy in rodent models	✓ Difficult transplantation due to tight capsule in large animals ✓ Systemic insulin release
**Omental pouch**	✓ Potential to accommodate large numbers of islets ✓ Rich vascularity ✓ Physiological insulin secretion	✓ No reports ✓ No clinical trials ✓ Possibility of risk associated with surgery including adhesion and ileus
**Mesentery**	✓ Rich vascularity ✓ Physiological insulin drainage	✓ Impossibility of graft removal without sacrificing intestinal tract
**Gastrointestinal tract**	✓ Rich vascularity ✓ Physiological insulin secretion ✓ Possibility of endoscopic approach	✓ Impossibility of graft removal without sacrificing intestinal tract
**Muscle and subcutaneous tissue**	✓ Easiest access with minimum invasion	✓ Poorest transplant efficacy ✓ Systemic insulin release
**Immune privilege site (brain, testis, eye, thymus)**	✓ Prevention, reduction or suppression of immunity	✓ Difficult clinical setting

**Table 2:** Outcomes of intrasplenic islet transplantation.

Authors [Reference]	Publication Year	Transplant Model	Comments
Kolb E et al. [89]	1977	Auto (dog)	✓ Achieved normoglycemia, but glucose tolerance was impaired
Feldman SD et al. [90]	1977	Auto (dog)	✓ Achieved normoglycemia, but glucose tolerance was impaired ✓ Implantation into splenic pulp
Gray BN et al. [92]	1979	Auto (dog)	✓ Response of insulin and glucagon to arginine stimulation ✓ Implantation into splenic pulp
Mehigan DG et al. [119]	1981	Auto (dog)	✓ Assessment of quality of collagenase
Andersson A et al. [120]	1981	Iso (mouse)	✓ Achieved normoglycemia after transplantation of 500 islets
Steffes MW et al. [121]	1981	Iso, allo (mouse)	✓ Minimum of 13 weeks of nearly normal glucose levels after receiving skin grafts and spleen cells
Du Toit DF et al. [115]	1982	Allo (dog)	✓ Extended survival, but normoglycemia not achieved
Janney CG et al. [116]	1982	Xeno (rat-to-mouse)	✓ Prolongation of more than up to 100 days graft survival using cultured islets and administration of anti-mouse and/or anti-rat lymphocyte sera
Andersson A. [118]	1982	Allo (mouse)	✓ Graft survival of several weeks with cultured islets but without immunosuppressants
Toledo-Pereyra LH et al. [103]	1983	Allo (dog)	✓ Graft using cryopreserved islets was not rejected for more than 60 days
Warnock GL et al. [122]	1983	Iso (dog)	✓ Five-month graft survival ✓ Implantation via splenic vein
Andersson A [123]	1983	Iso (mouse)	✓ Implantation of 500 islets was sufficient to achieve normoglycemia, while implantation of 150 islets was not
Merrell RC et al. [93,94]	1985	Auto (dog)	✓ Achieved normoglycemia ✓ Implantation via splenic vein
Kneteman NM et al. [124]	1985	Allo (dog)	✓ Prolongation of graft survival (approximately 20 days) using cyclosporine
Gray DW et al. [113]	1986	Auto (monkey)	✓ Achieved normoglycemia for 6 months ✓ First report of monkey model
Gores PF et al. [95]	1987	Auto (dog)	✓ Achieved normoglycemia for more than 30 days
Kneteman NM et al. [109]	1987	Allo (dog)	✓ Achieved normoglycemia for more than 100 days using cyclosporine
Hayek A et al. [125]	1988	Iso (rat)	✓ Partially achieved normoglycemia by transplantation of 1000 neonatal islets
Sutton R et al. [114]	1989	Auto (monkey)	✓ Achieved normoglycemia with reduced insulin response
Evans MG et al. [96]	1989	Auto (dog)	✓ The normoglycemic rate was 90% at 1 month after transplantation
van der Vliet JA et al. [97,98]	1989	Auto (dog)	✓ Normoglycemic rate 63%
Warnock GL et al. [99]	1990	Auto (dog)	✓ Normoglycemic rate 63% ✓ Comparison between splenic vein and pulp as the route of transplantation. Intravenous route superior (normoglycemia rate 86% vs. 33%)
Ziegler B et al. [126]	1990	Iso (rat)	✓ Achieved normoglycemia by transplantation of 1,200 islets
Korsgren O et al. [127]	1990	Iso (mouse)	✓ Achieved normoglycemia by transplantation of 500 islets
Scharp DW et al. [100]	1992	Auto (dog)	✓ Normoglycemic rats 86% at 1 year after transplantation
Motojima K et al. [101]	1992	Auto (dog)	✓ Normoglycemia not achieved
Marchetti P et al. [102]	1993	Auto (dog)	✓ Normoglycemic rate 90%, and decreased to 71% at 1 year after transplantation
Ao Z et al. [56]	1993	Auto (dog)	✓ Normoglycemic rate 67%
Yakimets WJ et al. [117]	1993	Allo (dog)	✓ Approximate 20 days graft survival using cyclosporine and rapamycin
Hesse UJ et al. [111]	1994	Auto (pig)	✓ The normoglycemic rate was 50%.
Eizirik DL et al. [128]	1997	Xeno, allo (human and mouse-to-nude mouse)	✓ Normoglycemia achieved by transplantation of 300 human islets into renal subcapsular space or 200 mouse islets into pulp of the spleen
Horton PJ et al. [77]	2000	Allo (dog)	✓ Normoglycemia achieved by pre-transplant irradiation of total lymphocytes and donor-specific bone marrow transplantation

**Table 3:** Efficacy of intrasplenic islet transplantation.

Authors [Reference]	Publication Year	Transplant model	Comments
Sutton R et al. [114]	1989	vs. Liver (auto, monkey)	✓ Intrasplenic transplantation showed no superiority over intraportal transplantation
Evans MG et al. [96]	1989	vs. Liver, kidney (auto, dog)	✓ Transplantation efficacy better in intrasplenic transplanted dog model: 90% achieved normoglycemia at 1 month, compared with 33% for intraportal and 0% for renal subcapsular
van der Vliet JA et al. [97,98]	1989	vs. Liver (auto, dog)	✓ Normoglycemic rate 63% for intrasplenic vs. 75% for intraportal
Warnock GL et al. [99]	1990	vs. Liver (auto, dog)	✓ Normoglycemic rate 63% for intrasplenic vs. 80% for intraportal ✓ Hyperglycemia after transplantation was less severe and onset was delayed
Scharp DW et al. [100]	1992	vs. Liver (auto, dog)	✓ Normoglycemic rate 86% for intrasplenic vs. 50% for intraportal at 1 year after transplantation
Motojima K et al. [101]	1992	vs. Liver (Auto, dog)	✓ Normoglycemia not achieved with either intrasplenic or intraportal transplantation
Ao Z et al. [56]	1993	vs. Omental pouch (auto, dog)	✓ Normoglycemic rate 67% for intrasplenic vs. 50% for intraomental transplantation
Hesse UJ et al. [111]	1994	vs. Liver (auto, pig)	✓ Normoglycemic rate 50% for intrasplenic vs. 25% for intraportal transplantation
Gustavson SM et al. [131]	2005	vs. Omental pouch (auto, dog)	✓ Transplantation efficacy better for intrasplenic versus intraomental pouch transplantation as assessed by glucose tolerance test
Stokes RA et al. [129]	2017	vs. Liver, kidney (Allo, pig)	✓ Allo-transplant model using fetal porcine islets. Transplantation efficacy was kidney > spleen > liver
Stokes RA et al. [130]	2017	vs. Liver, kidney (iso, mouse) vs. Liver, kidney, portal vein, muscle (xeno, human-to-SCID mouse)	✓ Iso: transplantation of 220–250 islets. Normoglycemia rate 100% in kidney, 29% in spleen, 0% in liver (subcapsular space was used in the spleen and liver transplant models) ✓ Xeno: transplantation of human 2000 islets. Normoglycemia rate 100% for kidney, 70% for muscle, and 60% for portal vein

## Abbreviations

HMGB1	high-mobility group box 1
IBMIR	instant blood-mediated inflammatory reaction
IL	interleukin
NOD	non-obese
Rrm2b	ribonucleoside-diphosphate reductase subunit M2 b
T-ALL	T-cell acute lymphoblastic leukemia
Tregs	regulatory T cells
